# A non-native fish species reaches the south-western European waters: the Atlantic croaker, *Micropogoniasundulatus* (Acanthuriformes, Sciaenidae) and its invasion history in Europe

**DOI:** 10.3897/BDJ.12.e120736

**Published:** 2024-05-14

**Authors:** Gustavo Freire de Carvalho-Souza, Cristóbal Lobato Gómez, Enrique González-Ortegón

**Affiliations:** 1 Instituto de Ciencias Marinas de Andalucía (ICMAN-CSIC), Puerto Real, Spain Instituto de Ciencias Marinas de Andalucía (ICMAN-CSIC) Puerto Real Spain; 2 Universidad de Cádiz, Instituto Universitario de Investigación Marina (INMAR), Campus de Excelencia Internacional/Global del Mar (CEI·MAR), Puerto Real, Spain Universidad de Cádiz, Instituto Universitario de Investigación Marina (INMAR), Campus de Excelencia Internacional/Global del Mar (CEI·MAR) Puerto Real Spain; 3 Agencia de Gestión Agraria y Pesquera de Andalucía, Sevilla, Spain Agencia de Gestión Agraria y Pesquera de Andalucía Sevilla Spain

**Keywords:** alien species, biological invasion, exotic species, first record, Iberian Peninsula, sciaenid fish

## Abstract

The Atlantic croaker *Micropogoniasundulatus*, a sciaenid fish native to the North Atlantic American coast, holds importance in recreational and commercial fisheries. Moreover, its potential as an invasive species should be noted, given its expansion and establishment in Atlantic European waters. This study reports its southernmost occurrence in Europe, in the Gulf of Cadiz. Morphological and molecular analysis confirmed its identity, revealing genetic similarities to US sequences. A comprehensive review of historical non-native distribution records underscored the species' expansion throughout European waters, suggesting human-mediated introduction. The escalating frequency of such arrivals emphasises the critical need for effective monitoring and management efforts in order to control non-native species in this region.

## Introduction

The phenomenon of biological invasions is universally acknowledged as an important component of human-induced environmental transformations, closely linked to the globalisation of the economy and the expansion of human populations (Hudgins et al. 2023). The recent upsurge in non-native marine and estuarine species introduction on a global scale (Occhipinti-Ambrogi 2007) has been predominantly attributed to human activities (e.g. marine traffic, pollution) and the ongoing global climate changes (Katsanevakis et al. 2014, González-Ortegón and Moreno-Andrés 2021). These intruded non-native species can exert significant influence on ecosystems, either by preying upon and/or competing with native species or by introducing parasites and pathogens that have the potential to jeopardise indigenous environments (Chalkowski et al. 2018, Cerveira et al. 2021, Ortega-Jiménez et al. 2024).

In the Gulf of Cadiz (GoC), the proliferation of non-native species (NNS) has steadily increased since 1980 (González-Ortegón et al. 2020a), thereby constituting a serious menace to the native biodiversity of this southernmost point of the Atlantic coast of Europe. This encompassing list includes various organisms, such as the mummichog *Fundulusheteroclitus* (Linnaeus, 1766), the Eastern mosquitofish, *Gambusiaholbrooki* Girard, 1859, the weakfish, *Cynoscionregalis* (Bloch & Schneider, 1801) (Morais and Teodósio 2016, Bañón et al. 2017), the African moonfish, *Selenedorsalis* (Gill, 1863) (Juárez et al. 2008), the sea slug *Polycerahedgpethi* Marcus, 1964 (Escribano-Álvarez and López-González 2015) and many decapod crustaceans, such as *Palaemonmacrodactylus* Rathbun, 1902 (Cuesta et al. 2004), *Penaeusmonodon* Fabricius, 1798, *Callinectessapidus* Rathbun, 1896 (González-Ortegón et al. 2020a), *Lysmatauncicornis* Holthuis & Maurin, 1952 (González-Ortegón et al. 2020b), *Portunussegnis* (Forskål, 1775) (de Carvalho-Souza et al. 2023b), *Penaeusnotialis* Pérez Farfante, 1967 (González-Ortegón et al. 2024) and Alpheuscf.lobidens (de Carvalho-Souza et al. 2024b).

Another non-native species reported in European waters is the Atlantic croaker, *Micropogoniasundulatus* (Linnaeus, 1766), a sciaenid fish. First observed near Ostend, Belgium, in 1998 (Eneman 1998), this species was later found in the Scheldt Estuary in 2001 and on the Dutch coast in 2003 (Rappé 2002, Stevens et al. 2004), with subsequent successive captures throughout these two decades in the Greater North Sea (Dekker et al. 2005, ICES 2006, Kerckhof 2007, Carl and Møller 2016, Gittenberger et al. 2017, Langeveld and Oosterbaan 2017, Andersen et al. 2021, ICES 2022a, GBIF 2023). Additionally, it is cited as an invasive species in the Chinese part of the Yellow Sea through aquaculture (Xiong et al. 2017), while in its native range, this species is distributed discontinuously throughout the Americas, from Massachusetts to Yucatán, USA and Mexico (Chao and Musick 1977).

The *Micropogoniasundulatus* is an euryhaline demersal inshore species that predominantly inhabits the bottom zones of mixed environments, including mud, sand and shell substrates, as well as areas with sponge and coral (Bearden 1964, Lankford et al. 1999). The early life stages of the Atlantic croaker utilise coastal and estuarine waters as a nursery area (Nixon and Jones 1997). These sciaenid fish are essentially bottom-dwelling predators, feeding on a diet that includes crustaceans, polychaetes, molluscs and small fish (Overstreet and Heard 1978, Froese and Pauly 2023). Due to their abundance, the species is one of the most important and traditional targets of commercial and recreational fisheries (Barbieri et al. 1997).

In this paper, we report the first occurrence of *M.undulatus* on the European south coast, near the mouth of the Guadalquivir Estuary in the Gulf of Cadiz, expanding its non-native range, previously restricted to the Greater North Sea. This represents the southernmost record of the species in the Eastern Atlantic at the moment.

Dekker et al. 2005, ICES 2006, Kerckhof 2007, Carl and Møller 2016, Gittenberger et al. 2017, Langeveld and Oosterbaan 2017, Andersen et al. 2021, ICES 2022a, GBIF 2023

## Material and Methods

### Sampling, morphological identification

A single specimen was caught with trammel nets by local fishermen (vessel Maria Mar Primera; ESP000024745) in the Gulf of Cadiz (SW Spain). This capture took place during a commercial fishing operation conducted near the mouth of the Guadalquivir River (36°38'35.2"N - 6°27'38.0"W), at a depth of 17 m. The specimen was measured (mm) and photographed during fishing landing and its previous identification was made using descriptions and key classifications (Chao 1978, Farrag 2022, de Carvalho-Souza et al. 2023a). Fin samples were collected for subsequent molecular identification.

### DNA extraction, amplification and sequencing

DNA was extracted from fin tissue using the Omega Bio-tek protocol (E.Z.N.A.® Tissue DNA Kit). Fragments of the mitochondrial gene cytochrome c oxidase subunit 1 (COI) were amplified with the primers FishF2 (5’ TCGACTAATCATAAAGATATCGGCAC 3’) and FishR2 (5’ ACTTCAGGGTGACCGAAGAATCAGAA 3’) (Ward et al. 2005). Polymerase chain reaction (PCR) for the sample comprised a total volume 12.5 μl, containing 1.25 μl of template DNA, 0.5 μM of the primers, 3.13 μl of Supreme NZYTaq 2x Green Master Mix (NZYTech) and ultrapure water up to 12.5 μl. The PCR cycles were as follows: an initial denaturation step at 95°C for 5 min, followed by 35 cycles of 95°C for 30 s, 53°C for 30 s, 72°C for 45 s and a final extension step at 72°C for 5 min (Bioer GeneExplorer™ PCR thermal cycler). The PCR product was run on 2% agarose gels stained with GreenSafe (NZYTech) and imaged under UV light to verify the amplicon size; then it was purified using magnetic beads (MagBind, Omega Bio-tek) prior to sequencing. Afterwards, the PCR product was bi-directionally sequenced on a ABI 3730xl DNA Analyzer (Applied Biosystems, USA), with the same primers as those used in the PCR amplification. Several sequencing runs were performed in order to improve the quality of the sequence data.

### Molecular identification

The COI partial sequence (582 bp) of one specimen was generated (Genank accession number OR906314; Suppl. material 1). The sample was identified to the lowest taxonomic level by comparing the consensus sequences obtained with the alignment of both forward and reverse sequences against the NCBI’s Nucleotide database using the Basic Local Alignment Search Tool (BLASTn) (Camacho et al. 2009) web server. The molecular identification was performed on the official Barcode of Life Database (BOLD) (http://v3.boldsystems.org/index.php/IDS_OpenIdEngine) to obtain the best fitting matching sequences.

### Review of non-native distribution

To review the non-native occurrence records of *M.undulatus*, we conducted a comprehensive bibliographic research, compiling and updating records. The search encompassed literature published from 1970 to November 2023, sourced from the Web of Science database, Scopus and Google Scholar. The search employed the following keywords (and/or): “*Micropogoniasundulatus*”, “*Percaundulata*”, “Atlantic croaker”, “first record”, “occurrence”, “range expansion”, “non-native”, “exotic”, “alien”. Additional bibliographic sources were obtained by reviewing the reference lists of the located publications. Furthermore, the information was cross-verified with reports from the ICES' Working Group on Introductions and Transfers of Marine Organisms (WGITMO) published from 1972 to 2022, data obtained from the GBIF database (https://www.gbif.es) and citizen-science platforms (e.g. iNaturalist.com, Waarneming.nl and Observadoresdelmar.es).

## Results

### Specimen examined

**Figure 1.**
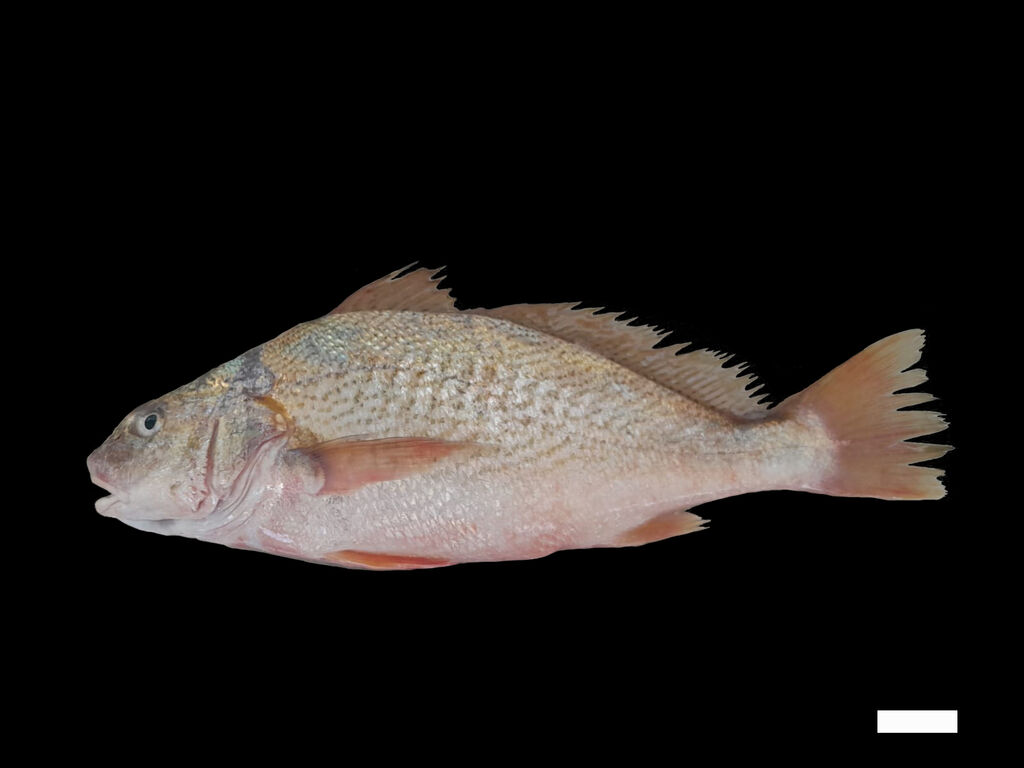
Specimen of *Micropogoniasundulatus* recorded in the Gulf of Cadiz, in July 2023. Scale bar: 5 cm.

The first-ever record of the Atlantic croaker *Micropogoniasundulatus* in the south-western Atlantic European waters is reported in this study, specifically near the mouth of the Guadalquivir Estuary within the Gulf of Cadiz. This specimen measured 41.5 cm, weighed 1.05 kg and was captured on 13 July 2023 (Table 1). The individual was sold in the fish market in Rota (Gulf of Cadiz), but it was photographed before the sale (Fig. 1).

### Molecular identification

Partial COI sequence obtained from the analysed specimen (OR906314, submitted to GenBank; https://www.ncbi.nlm.nih.gov/nuccore/OR906314) shows a 99.31% similarity to 28 sequences (Suppl. material 2) from *M.undulatus* specimens collected in the United States. Moreover, it displays a similarity range of 99.3% to 99.14% with six other sequences deposited in GenBank. These sequences originated from specimens collected in several locations along the coast of the United States, such as the Gulf of Mexico, Massachusetts, Texas, Maryland, Alabama and Florida.

### Non-native distribution

The compilation conducted here demonstrates at least 23 confirmed records of Atlantic croaker occurrences in European Atlantic waters (Table 2). Historical records of *M.undulatus* occurrences depict the three phases of the invasion process (see references in Table 2): arrival between 1998 and 2002 in Belgian waters, initial expansion with records in Dutch waters from 2003, establishment during this decade along the Belgian and Dutch coasts and subsequent north-south expansion across the Greater North Sea ecoregion (ICES 2022b), being recorded in Danish waters in 2008 and 2018 and in the English Channel in 2020. The present record in the Gulf of Cadiz demonstrates a new arrival in its southernmost record.

## Discussion

In the Gulf of Cadiz, a significant number of non-native species (NNS) is attributed to the northward expansion range in response to warming conditions (González-Ortegón et al. 2020a, González-Ortegón et al. 2020b). However, it is crucial to acknowledge that the introduction of species through human-mediated means, particularly via vessels, also plays a important role on the local biodiversity changes (González-Ortegón and Moreno-Andrés 2021).

To date, including the present finding, two non-native sciaenid species that have possibly been introduced in ballast water of ships are known along the GoC. One of them, *C.regalis*, has been established since 2011 in the Gulf of Cadiz and 2014 in the Sado Estuary through multiple and independent events, possibly dispersing to adjacent areas (Morais et al. 2017). However, another study suggested that its origin is due to expansions of the first introduction in the Iberian Peninsula, in the Guadalquivir Estuary (Bañón et al. 2018). This sciaenid is native to the western Atlantic and its distribution ranges from northern Florida (USA) to Nova Scotia (Canada) (Froese and Pauly 2023). It was introduced in Europe at the last decade and, currently, it has become ubiquitous in almost the entire Atlantic part of the Iberian Peninsula (Bañón et al. 2018). Currently, this species has become a target for local artisanal fisheries along various Iberian coastal and estuarine waters, such as Galician waters, Ría Formosa Lagoon, Guadalquivir, Guadiana, Sado, Tagus and Mira Rivers (Béarez et al. 2016, Bañón et al. 2017, Morais et al. 2017).

The present observation of *M.undulatus* represents the first record for the Gulf of Cadiz and the second ecoregion in European waters (Stevens et al. 2004). The initial occurrences were documented in Belgium in 1998 and 2001, specifically near Ostend and the Scheldt Estuary (Stevens et al. 2004). Subsequently, a 16 cm individual was captured in the eastern Wadden Sea between Lauwersoog and Delfzijl in 2003 (Dekker et al. 2005). In 2004, two other immature female specimens of similar sizes were captured in the North Sea Channel and examined at the Netherlands Institute for Fisheries Research (RIVO) (see Table 2; Dekker et al. (2005)). These authors propose that these individuals originated from the expansion of the initial introduction, as only young-of-the-year were caught, displaying no signs of growth retardation related to stressful transport in ballast tanks. Another specimen was captured in Belgian coastal waters in 2005 (ICES 2006) and, from 2006, in various locations in the Netherlands, including the port areas of Rotterdam (Dintelhaven), Amsterdam, Reimerswaal, Velsen and Zaanstad (Meyling 2008, GBIF 2023), indicating its establishment in these areas. In 2008, an individual measuring 25 cm was reported for the first time in Danish waters, north of Skagen (Carl and Møller 2016). Throughout the following decade, several new records were reported in these locations, featuring increasingly larger individuals. For instance, a specimen of 43 cm captured in the North Sea Canal near the Zaan in November 2016 (Janssen 2016; Table 2). In October 2020, a specimen measuring 28.5 cm was caught 4 miles (ca. 6 km) southeast off Plymouth, Cornwall/Devon, England. This young individual was identified by experts upon discovery at the Plymouth fish market (ICES 2022a).

The absence of information regarding whether the specimen found here indicates a population spread from the Belgian/Dutch waters, where this fish may have established, introduces a degree of uncertainty about its origin. Nevertheless, it is unlikely that individuals introduced into the European North would have migrated southwards to colonise the Gulf of Cadiz without being observed in ecosystems situated between these regions.

**Figure 2.**
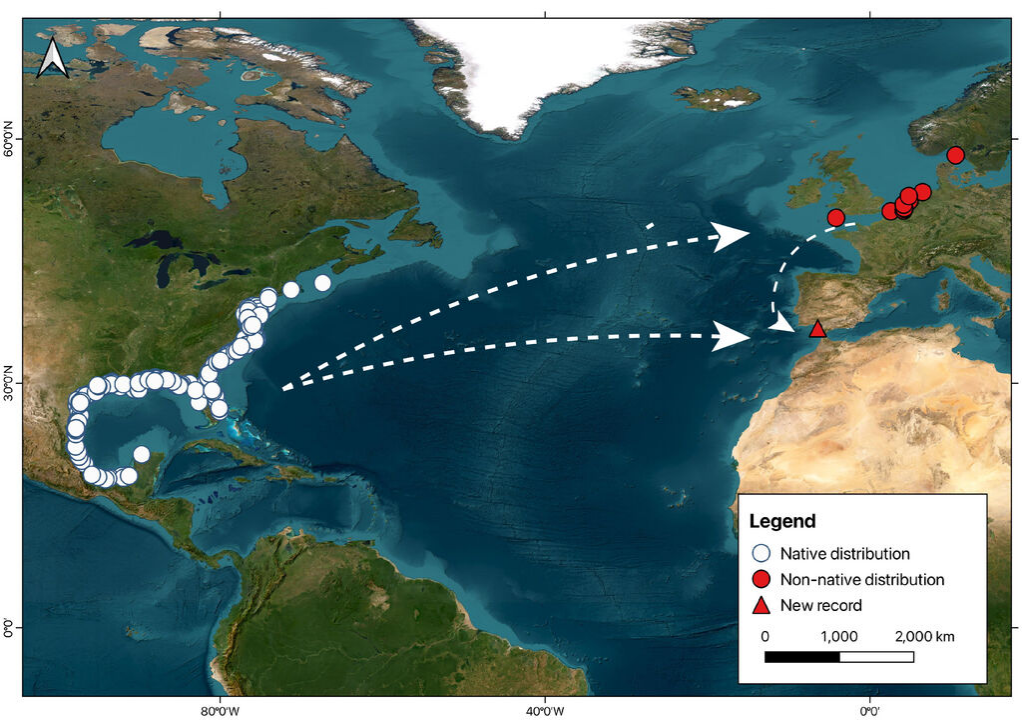
Records of the Atlantic croaker, *Micropogoniasundulatus*, in the Atlantic waters are depicted on the map. White dots indicate native locations of *M.undulatus* in the western Atlantic Ocean. Red dots indicate previous non-native locations of *M.undulatus* in the Greater North Sea, while the red triangle marker indicates the location of the *M.undulatus* recorded in the Gulf of Cadiz during this study (Rota, near the mouth of the Guadalquivir Estuary). White arrows indicate the probable route of arrival through ballast water.

The likely arrival route was through ballast water, given the substantial distances from its origin zone and prior invasions (Fig. 2). In the Greater North Sea, there are two of the largest ports in Europe (Rotterdam and Antwerp), both receiving large amounts of ballast water from foreign sources. Concerning the Gulf of Cadiz, a probable route involves ballast water from ships connecting important ports: the Port of Seville (in the Guadalquivir Estuary), Port of Cadiz (40 km from the Guadalquivir mouth), Port of Algeciras and Tangier Med (both in the Strait of Gibraltar). This scenario indicates that this may be yet another rare example of a fish species introduced through multiple and independent introduction events, with records in two ecoregions with high propagule pressure in Europe – the Greater North Sea and the Gulf of Cadiz and within those regions (southern zone of the Bay of Biscay and Iberian Peninsula ecoregion; ICES (2020)).

The capture of a single individual suggests that the Atlantic croakers in the Gulf of Cadiz may not be fully established, in contrast to the robust growth and active fishing observed for *C.regalis* (de Carvalho-Souza et al. 2024a). Furthermore, the growth of the *M.undulatus* population might face limitations due to competition for space and resources with other sciaenids, both non-native species (specifically, *C.regalis*) and native species, such as meagre, *Argirosomusregius* (Asso, 1801).

On the other hand, the Guadalquivir Estuary is recognised as an important nursery area and essential fish habitat for numerous commercial species, such as the European anchovy (*Engraulisencrasicolus*), sardine (*Sardinapilchardus*), seabass (*Dicentrarchuslabrax*),and shrimps (*Palaemon* spp.) (de Carvalho-Souza et al. 2019, González-Ortegón et al. 2023). This environment can offer ideal conditions for the expansion and establishment of the species.

Given these favourable conditions in the Estuary, it is necessary to initiate proactive control measures and potential eradication efforts early, as the species is not yet completely established in the Gulf of Cadiz. This observation underscores the continued importance of monitoring the arrival and dispersion of NNS in the Gulf of Cadiz, as well as the need for effective measures in ecosystem-based NNS management.

## Data resources

The data underpinning the analysis reported in this paper are deposited at GBIF, the Global Biodiversity Information Facility, https://ipt.pensoft.net/resource?r=micropogonias_undulatus_occurrence_records_europe.

## Supplementary Material

E5E7E575-29FA-508D-A153-974AED67A650 10.3897/BDJ.12.e120736.suppl1 Supplementary material 1 COI partial sequence (OR906314) from GenBank flatfile format Data type COI partial sequence (582 bp) File: oo_1029390.txt https://binary.pensoft.net/file/1029390 Gustavo F. de Carvalho-Souza, Cristóbal Lobato Gómez, and Enrique González-Ortegón

6B30164B-6301-5687-AB43-5BBC67626DDC 10.3897/BDJ.12.e120736.suppl2 Supplementary material 2 Published records in GenBank for *Micropogoniasundulatus*: cytochrome c oxidase subunit I (COI) gene Data type occurrences Brief description Published records in GenBank of *Micropogoniasundulatus* cytochrome c oxidase subunit I (COI) gene. File: oo_1029401.docx https://binary.pensoft.net/file/1029401 Gustavo F. de Carvalho-Souza, Cristóbal Lobato Gómez, and Enrique González-Ortegón

## Figures and Tables

**Table 1.** Measurements and counts of specimens of *Micropogoniasundulatus*: Belgian coast, Schelde Estuary (Stevens et al. 2004) and Gulf of Cadiz (present study).

Measure (mm) or count	Belgian coast (Stevens et al. 2004)	Schelde (Stevens et al. 2004)	Gulf of Cadiz (Present study)
Total length	146	132.7	410.5
Fin rays			
dorsal	IX+I, 26	IX+I, 29	X+I, 29
anal	II, 8	II, 7	II, 8
pelvic	R4, L5	6	6
pectoral	17	17	17
Weight (g)	/	20.8	1050

**Table 2.** Historical occurrence records of Atlantic croakers, *Micropogoniasundulatus* in its European non-native range. Acronym: N - Number of individuals; TL - Total length (cm). *Coordinates estimated from record description.

**Year**	**N**	**Locality**	**Latitude**	**Longitude**	**TL (cm)**	**Reference**
August 1998	1	Westerschelde Estuary, Belgian waters	51°13’N	2°55’E	14.6	Eneman (1998); Rappé (2002)
October 2001	1	Scheldt Estuary, Belgian waters	51°22’N	4°14’E	13.2	Stevens et al. (2004)
October 2003	1	Wadden Sea, Dutch waters	53°47.57’N	6°47.91’E	16	Dekker et al. (2005)
October 2004	2	North Sea Canal	52°25.50’N	4°46.08’E	16.4-17.2	Dekker et al. (2005)
August 2005	1	Belgian coastal waters	-	-	19	ICES (2006)
2006	1	Rotterdam, Dutch waters	51°87’N	4°25’E	-	Meyling (2008)
2007	5	Amsterdam, Dutch waters	52°41’N	4°83’E	-	GBIF (2023)
2007	2	Reimerswaal, Dutch waters	51°41’N	4°83’E	-	GBIF (2023)
2007	2	Velsen, Dutch waters	52°46’N	4°64’E	-	GBIF (2023)
2007	2	Zaanstad, Dutch waters	52°41’N	4°85’E	-	GBIF (2023)
December 2008	1	Amsterdam, Dutch waters	52°40.98’N	4°84.55’E	-	Bron (2008). Waarneming.nl -citizen science platform
December 2008	1	North of Skagen, Danish waters	58°00’N*	10°55’E*	25	Carl and Møller (2016)
September 2013	1	Texel-Mokbaai- Estuary (North Holland)	52°99.82’N	4°77.59’E	18	Witte (2013). Waarneming.nl -citizen science platform
August 2014	1	Scheldt-Rhine Canal	51°41.92’N	4°23.54’E	26	Koens (2014). Waarneming.nl -citizen science platform
September 2014	1	Texel-Mokbaai- Estuary (North Holland)	52°99.70’N	4°77.49’E	-	Witte (2014). Waarneming.nl -citizen science platform
2014	1	Scheldt-Rhine Canal	51°51’N*	4°21’E*	-	Delft et al. (2015)
August 2015	1	Europoort/Rotterdam, Dutch waters	51°95’N*	4°14’E*	30.5	Secretariaat NCRZ (2016)
September 2016	1	North Sea Canal	52°25’N	4°46’E	43	Janssen (2016)
September 2017	1	Rotterdam, Dutch waters	51°87.10’N	4°25.40’E	37	Langeveld and Oosterbaan (2017)
2018	1	Nordsøen/Skagerrak, Danish waters	-	-	-	Andersen et al. (2021)
September 2020	2	Texel- Mokbaai- NIOZ fyke	52°99.68’N	4°77.82’E	33	Mosk (2020). Waarneming.nl -citizen science platform
October 2020	1	off Plymouth	50°31’N*	-4°17’E	28.5	ICES (2022a)
July 2023	1	Gulf of Cadiz, Spain	36°38’35.2’N	6°27’38.0’W	41.5	Present record
