# Supplementary material for: A non-native fish species reaches the south-western European waters: the Atlantic croaker, Micropogoniasundulatus (Acanthuriformes, Sciaenidae) and its invasion history in Europe
Source: Biodivers Data J. 2024 May 14;12:e120736. doi: 10.3897/BDJ.12.e120736 (PMC11109509; doi:10.3897/BDJ.12.e120736)
Supplement: Supplementary material 2 — Published records in GenBank for Micropogoniasundulatus: cytochrome c oxidase subunit I (COI) gene [file bdj-12-e120736-s002.docx]

**A non-native fish species reaches the southwestern European waters: the Atlantic croaker, *Micropogonias undulatus* (Acanthuriformes, Sciaenidae), and its invasion history in Europe**

**Gustavo F. de Carvalho-Souza^1,2*^, Cristóbal Lobato Gómez^3^, and Enrique González-Ortegón^1*^**

^1^ Instituto de Ciencias Marinas de Andalucía (ICMAN-CSIC), Campus Universitario Río San Pedro, 11519, Puerto Real, Cádiz, Spain

^2^ Universidad de Cádiz, Campus de Excelencia Internacional/Global del Mar (CEI·MAR), 11510, Puerto Real (Cádiz), Spain

^3^ Agencia de Gestión Agraria y Pesquera de Andalucía, c/ Bergantín 39, 41012 Sevilla, Spain

Corresponding author: [gustavo.souza@csic.es](mailto:gustavo.souza@csic.es); [gustavofcsouza@gmail.com](mailto:gustavofcsouza@gmail.com)

**Supplementary Information**

**Table S1**. Records published in Genbank of *Micropogonias undulatus* cytochrome c oxidase subunit I (COI) gene.

| **GenBank accession number** | **Species Identification (Definition)** | **Locality** | **Similarity (%)** | **Authors** | **Reference** |
| --- | --- | --- | --- | --- | --- |
| [MH379059](http://www.ncbi.nlm.nih.gov/nuccore/MH379059) | *Micropogonias undulatus* isolate RF49 cytochrome c oxidase subunit I (COI) gene, partial cds; mitochondrial | USA | 99.31 | Stoeckle, M.Y. and Das Mishu, M. | Stoeckle et al., 2018 |
| [MH379067](http://www.ncbi.nlm.nih.gov/nuccore/MH379067) | *Micropogonias undulatus* isolate RF57 cytochrome c oxidase subunit I (COI) gene, partial cds; mitochondrial | Massachusetts, USA | 99.31 | Stoeckle, M.Y. and Das Mishu, M. | Stoeckle et al., 2018 |
| [MH379069](http://www.ncbi.nlm.nih.gov/nuccore/MH379069) | *Micropogonias undulatus* isolate RF59 cytochrome c oxidase subunit I (COI) gene, partial cds; mitochondrial | Massachusetts, USA | 99.31 | Stoeckle, M.Y. and Das Mishu, M. | Stoeckle et al., 2018 |
| [MH378574](http://www.ncbi.nlm.nih.gov/nuccore/MH378574) | *Micropogonias undulatus* voucher USNM:FISH:433252 cytochrome oxidase subunit 1 (COI) gene, partial cds; mitochondrial | USA | 99.31 | Redmond, N., Pitassy, D. and Trizna, M. | Smithsonian, 2018 |
| [MH378587](http://www.ncbi.nlm.nih.gov/nuccore/MH378587) | *Micropogonias undulatus* voucher USNM:FISH:433266 cytochrome oxidase subunit 1 (COI) gene, partial cds; mitochondrial | USA | 99.31 | Redmond, N., Pitassy, D. and Trizna, M. | Smithsonian, 2018 |
| [MW535333](http://www.ncbi.nlm.nih.gov/nuccore/MW535333) | *Micropogonias undulatus* isolate 59553 cytochrome c oxidase subunit I (COX1) gene, partial cds; mitochondrial | East Matagorda Bay, Texas, USA | 99.31 | Williford, D., Anderson, J. and Pineda, K. | Williford, Anderson, and Pineda, 2017 |
| [MW535332](http://www.ncbi.nlm.nih.gov/nuccore/MW535332) | Micropogonias undulatus isolate 59561 cytochrome c oxidase subunit I (COX1) gene, partial cds; mitochondrial | East Matagorda Bay, Texas, USA | 99.31 | Williford, D., Anderson, J. and Pineda, K. | Williford, Anderson, and Pineda, 2017 |
| [MW535331](http://www.ncbi.nlm.nih.gov/nuccore/MW535331) | *Micropogonias undulatus* isolate 59550 cytochrome c oxidase subunit I (COX1) gene, partial cds; mitochondrial | East Matagorda Bay, Texas, USA | 99.31 | Williford, D., Anderson, J. and Pineda, K. | Williford, Anderson, and Pineda, 2017 |
| [MW535329](http://www.ncbi.nlm.nih.gov/nuccore/MW535329) | *Micropogonias undulatus* isolate 59562 cytochrome c oxidase subunit I (COX1) gene, partial cds; mitochondrial | East Matagorda Bay, Texas, USA | 99.31 | Williford, D., Anderson, J. and Pineda, K. | Williford, Anderson, and Pineda, 2017 |
| [MW535328](http://www.ncbi.nlm.nih.gov/nuccore/MW535328) | *Micropogonias undulatus* isolate 59267 cytochrome c oxidase subunit I (COX1) gene, partial cds; mitochondrial | East Matagorda Bay, Texas, USA | 99.31 | Williford, D., Anderson, J. and Pineda, K. | Williford, Anderson, and Pineda, 2017 |
| [MW535327](http://www.ncbi.nlm.nih.gov/nuccore/MW535327) | *Micropogonias undulatus* isolate 59564 cytochrome c oxidase subunit I (COX1) gene, partial cds; mitochondrial | East Matagorda Bay, Texas, USA | 99.31 | Williford, D., Anderson, J. and Pineda, K. | Williford, Anderson, and Pineda, 2017 |
| [MW535326](http://www.ncbi.nlm.nih.gov/nuccore/MW535326) | *Micropogonias undulatus* isolate 59266 cytochrome c oxidase subunit I (COX1) gene, partial cds; mitochondrial | Galveston Bay, Texas, USA | 99.31 | Williford, D., Anderson, J. and Pineda, K. | Williford, Anderson, and Pineda, 2017 |
| [MW535325](http://www.ncbi.nlm.nih.gov/nuccore/MW535325) | *Micropogonias undulatus* isolate 59570 cytochrome c oxidase subunit I (COX1) gene, partial cds; mitochondrial | Matagorda Bay (Carancahua Bay), Texas, USA | 99.31 | Williford, D., Anderson, J. and Pineda, K. | Williford, Anderson, and Pineda, 2017 |
| [MW535324](http://www.ncbi.nlm.nih.gov/nuccore/MW535324) | *Micropogonias undulatus* isolate 59284 cytochrome c oxidase subunit I (COX1) gene, partial cds; mitochondrial | Galveston Bay, Texas, USA | 99.31 | Williford, D., Anderson, J. and Pineda, K. | Williford, Anderson, and Pineda, 2017 |
| [MW535323](http://www.ncbi.nlm.nih.gov/nuccore/MW535323) | *Micropogonias undulatus* isolate 59576 cytochrome c oxidase subunit I (COX1) gene, partial cds; mitochondrial | Matagorda Bay (Carancahua Bay), Texas, USA | 99.31 | Williford, D., Anderson, J. and Pineda, K. | Williford, Anderson, and Pineda, 2017 |
| [MW535322](http://www.ncbi.nlm.nih.gov/nuccore/MW535322) | *Micropogonias undulatus* isolate 59569 cytochrome c oxidase subunit I (COX1) gene, partial cds; mitochondrial | Matagorda Bay (Carancahua Bay), Texas, USA | 99.31 | Williford, D., Anderson, J. and Pineda, K. | Williford, Anderson, and Pineda, 2017 |
| [MW535330](http://www.ncbi.nlm.nih.gov/nuccore/MW535330) | *Micropogonias undulatus* isolate 59549 cytochrome c oxidase subunit I (COX1) gene, partial cds; mitochondrial | East Matagorda Bay, Texas, USA | 99.31 | Williford, D., Anderson, J. and Pineda, K. | Williford, Anderson, and Pineda, 2017 |
| [JQ841938](http://www.ncbi.nlm.nih.gov/nuccore/JQ841938) | *Micropogonias undulatus* voucher FCC8067 cytochrome oxidase subunit 1 (COI) gene, partial cds; mitochondrial | Florida, Brevard County, USA | 99.31 | Weigt, L. A., et al. | Weigt et al., 2012 |
| [JQ841936](http://www.ncbi.nlm.nih.gov/nuccore/JQ841936) | *Micropogonias undulatus* voucher FCC8068 cytochrome oxidase subunit 1 (COI) gene, partial cds; mitochondrial | Florida, Brevard County, USA | 99.31 | Weigt, L. A., et al. | Weigt et al., 2012 |
| [KC015693](http://www.ncbi.nlm.nih.gov/nuccore/KC015693) | *Micropogonias undulatus* voucher 07-106 cytochrome oxidase subunit 1 (COI) gene, partial cds; mitochondrial | Massachusetts, USA | 99.31 | McCusker, M.R., Denti, D., Van Guelpen, L., Kenchington, E. and Bentzen, P. | McCusker et al., 2013 |
| [KX459327](http://www.ncbi.nlm.nih.gov/nuccore/KX459327) | *Micropogonias undulatus* cytochrome oxidase subunit 1 (COI) gene, partial cds; mitochondrial | Maryland, Chesapeake, Bay, USA | 99.31 | Aguilar, R. et al. | Aguilar et al., 2017 |
| [KF461203](http://www.ncbi.nlm.nih.gov/nuccore/KF461203) | *Micropogonias undulatus* voucher FDA 225 cytochrome oxidase subunit 1 (COI) gene, partial cds; mitochondrial | Alabama, USA | 99.31 | Handy, S. M. et al. | Handy et al. 2011 |
| [KX163997](http://www.ncbi.nlm.nih.gov/nuccore/KX163997) | *Micropogonias undulatus* cytochrome oxidase subunit I (COI) gene, partial cds; mitochondrial | USA | 99.31 | Joseph, A. and Ehsan, H. | Joseph, and Ehsan, 2016 |
| [JQ841939](http://www.ncbi.nlm.nih.gov/nuccore/JQ841939) | *Micropogonias undulatus* voucher FCC8066 cytochrome oxidase subunit 1 (COI) gene, partial cds; mitochondrial | Florida, Brevard County, USA | 99.31 | Weigt, L. A., et al. | Weigt et al., 2012 |
| [HQ024967](http://www.ncbi.nlm.nih.gov/nuccore/HQ024967) | *Micropogonias undulatus* voucher FDA 2-23 cytochrome oxidase subunit 1 (COI) gene, partial cds; mitochondrial | USA | 99.31 | Handy, S. M. et al. | Handy et al. 2011 |
| [HQ024966](http://www.ncbi.nlm.nih.gov/nuccore/HQ024966) | *Micropogonias undulatus* voucher FDA 3-23 cytochrome oxidase subunit 1 (COI) gene, partial cds; mitochondrial | USA | 99.31 | Handy, S. M. et al. | Handy et al. 2011 |
| [HQ024965](http://www.ncbi.nlm.nih.gov/nuccore/HQ024965) | *Micropogonias undulatus* voucher FDA 4-23 cytochrome oxidase subunit 1 (COI) gene, partial cds; mitochondrial | USA | 99.31 | Handy, S. M. et al. | Handy et al. 2011 |
| [HQ024968](http://www.ncbi.nlm.nih.gov/nuccore/HQ024968) | *Micropogonias undulatus* voucher FDA 1-23 cytochrome oxidase subunit 1 (COI) gene, partial cds; mitochondrial | USA | 99.31 | Handy, S. M. et al. | Handy et al. 2011 |
| [KP722740](http://www.ncbi.nlm.nih.gov/nuccore/KP722740) | *Micropogonias undulatus* isolate CN732 cytochrome oxidase subunit 1 (COI) gene, partial cds; mitochondrial | USA | 99.3 | Lo, P.C., Liu, S.H., Chao, N.L., Nunoo, F.K., Mok, H.K. and Chen, W.J. | Lo et al., 2015 |
| [MH378539](http://www.ncbi.nlm.nih.gov/nuccore/MH378539) | *Micropogonias undulatus* voucher USNM:FISH:433207 cytochrome oxidase subunit 1 (COI) gene, partial cds; mitochondrial | USA | 99.28 | Redmond, N., Pitassy, D. and Trizna, M. | Smithsonian, 2018 |
| [JN021309](http://www.ncbi.nlm.nih.gov/nuccore/JN021309) | *Micropogonias undulatus* voucher Micropogonias undulatus1 cytochrome oxidase subunit 1 (COI) gene, partial cds; mitochondrial | USA | 99.14 | Shen, Y. and Ishida, M. | Shen, and Ishida, 2016 |
| [MH378515](http://www.ncbi.nlm.nih.gov/nuccore/MH378515) | *Micropogonias undulatus* voucher USNM:FISH:433173 cytochrome oxidase subunit 1 (COI) gene, partial cds; mitochondrial | USA | 99.14 | Redmond, N., Pitassy, D. and Trizna, M. | Smithsonian, 2018 |
| [MW535321](http://www.ncbi.nlm.nih.gov/nuccore/MW535321) | *Micropogonias undulatus* isolate 59255 cytochrome c oxidase subunit I (COX1) gene, partial cds; mitochondrial | Galveston Bay, Texas, USA | 99.14 | Williford, D., Anderson, J. and Pineda, K. | Williford, Anderson, and Pineda, 2017 |
| [KF930131](http://www.ncbi.nlm.nih.gov/nuccore/KF930131) | *Micropogonias undulatus* voucher KUT 5435 cytochrome oxidase subunit 1 (COI) gene, partial cds; mitochondrial | Gulf of Mexico, USA | 99.14 | Bentley, A.C. and Wiley, E.O | Bentley and Wiley, 2014 |
| [OR906314](https://www.ncbi.nlm.nih.gov/nuccore/OR906314) | *Micropogonias undulatus* isolate Mundu-2 cytochrome c oxidase subunit I (COX1) gene, partial cds; mitochondrial* | Gulf of Cadiz Spain | - | de Carvalho-Souza, G.F. and González-Ortegón, E. | This study |

References

Aguilar, R., Ogburn, M.B., Driskell, A.C., Weigt, L.A., Groves, M.C., Hines, A.H. 2017. Gutsy genetics: identification of digested piscine prey items in the stomach contents of sympatric native and introduced warmwater catfishes via DNA barcoding. Environ Biol Fish 100, 325–336. <https://doi.org/10.1007/s10641-016-0523-8>

Bentley, A.C. and Wiley, E.O. 2014. University of Kansas Biodiversity Institute barcoding initiative. <https://www.ncbi.nlm.nih.gov/nuccore/KF930131>

Handy, S.M., Deeds, J.R., Ivanova,N.V., Hebert, P.D., Hanner, R.H., Ormos, A., Weigt, L.A., Moore, M.M., Yancy, H.F. 2011. A single-laboratory validated method for the generation of DNA barcodes for the identification of fish for regulatory compliance. J AOAC Int. Jan-Feb;94(1):201-10. PMID: 21391497.

Joseph, A. and Ehsan, H. 2016. Biology, University of Houston-Victoria. <https://www.ncbi.nlm.nih.gov/nuccore/KX163997>

Lo, P.C., Liu, S.H., Chao, N.L., Nunoo, F.K., Mok, H.K., Chen, W.J. 2015. A multi-gene dataset reveals a tropical New World origin and Early Miocene diversification of croakers (Perciformes: Sciaenidae). Mol Phylogenet Evol. Jul; 88:132-43. <https://doi.org/10.1016/j.ympev.2015.03.025>

McCusker, M.R., Denti, D., Van Guelpen, L., Kenchington, E., Bentzen, P. 2013. Barcoding Atlantic Canada's commonly encountered marine fishes. Mol Ecol Resour. Mar;13(2):177-88. <https://doi.org/10.1111/1755-0998.12043>

Shen, Y. and Ishida, M. 2016. Office of Regulatory Affairs, Food and Drug Administration. <https://www.ncbi.nlm.nih.gov/nuccore/JN021309>

Smithsonian, 2018. DNA barcoding fish from US East Coast. Accession: PRJNA437661 ID: 437661 [dataset] (1970), omics_ena_project, V1. <https://www.ebi.ac.uk/ena/browser/view/PRJNA437657>

Stoeckle, M.Y., Das Mishu, M., Charlop-Powers, Z. 2018. GoFish: A versatile nested PCR strategy for environmental DNA assays for marine vertebrates. PLoS ONE 13(12): e0198717. <https://doi.org/10.1371/journal.pone.0198717>

Weigt, L.A., Baldwin, C.C., Driskell, A., Smith, D.G., Ormos, A., Reyier, E.A. 2012. Using DNA Barcoding to Assess Caribbean Reef Fish Biodiversity: Expanding Taxonomic and Geographic Coverage. PLoS ONE 7(7): e41059. <https://doi.org/10.1371/journal.pone.0041059>

2021. Identification of three common species of Sciaenidae (Red Drum *Sciaenops ocellatus*, Atlantic Croaker *Micropogonias undulatus*, and Spot Croaker Leiostomus xanthurus) using morphological characters and mitochondrial DNA sequences. <https://www.ncbi.nlm.nih.gov/nuccore/MW535331>
